# Experimental study on evaluation of density, P-wave velocity, thermal conductivity, and thermal diffusion coefficient of granite after thermal treatments by using PCA

**DOI:** 10.1038/s41598-024-58519-4

**Published:** 2024-04-02

**Authors:** Xinghui Wu, Changfu Huang, Peng Li, Shuailong Zhang, Zhe Xu

**Affiliations:** 1 School of City and Architecture Engineering, Zaozhuang University, Zaozhuang, 277160 Shandong China; 2 China Railway 15th Bureau Group Co. Ltd., Shanghai, 200070 China; 3 School of Civil and Resource Engineering, University of Science and Technology Beijing, Beijing, 100083 China

**Keywords:** Granite, PCA, Physical parameters, Parameter index, Thermal conductivity, Geology, Mineralogy

## Abstract

Temperature significantly influences the physical parameters of granite, resulting in variations in the rock's thermal conductivity. In order to examine the impact of changes in multiple physical parameters of granite at different temperatures on the thermal conductivity of rocks, Principal Component Analysis (PCA) was employed to determine the correlation between granite at different temperatures and various physical parameters, including density (*ρ*), P-wave velocity (*P*), thermal conductivity (*K*_*T*_), and thermal diffusion coefficient (*K*_*D*_). Utilizing the linear contribution rate, a single indicator '*y*' was derived to comprehensively represent the thermal conductivity of rocks. Research findings indicate that within the temperature range of 150–450 °C, the '*y*'-value is relatively high, signifying favorable thermal conductivity of the rock. Notably, longitudinal wave velocity demonstrates higher sensitivity to temperature changes compared to other physical parameters.

## Introduction

With the depletion of resources in the shallow section of the earth, numerous studies concentrate on the properties of deep rock^1–3^. The effects of temperatures on the physical–mechanical characteristics of granite have been extensively explored through numerous experiments^3–5^. Several studies^6–9^ identify the formation of microcracks in granite as the primary cause of heat degradation. Chaki et al.^10^. conducted examinations on granite's porosity, permeability, and P-wave velocity following high-temperature treatment (105–600 °C), revealing an increase in permeability and P-wave velocities with rising temperature, while porosity decreases. Chen et al.^11^. investigated the permeability of Beishan granite after high-temperature treatment (100–800 °C), highlighting the linear shift in volume strain of microcracks that impacts permeability. Liu et al.^12^. explored the mechanical characteristics of Qinling granite after high-temperature treatment (25–1000 °C), observing a decline in uniaxial compressive strength and tensile strength with increasing temperatures. Similarly, Yang et al.^13^. conducted uniaxial compression studies on granite after high-temperature treatment (25–1000 °C), revealing a rise in peak strength, crack damage stress, and elastic modulus between 25 and 300 °C, but a reverse trend between 300 and 800 °C.

The thermal conductivity of granite changes under the influence of temperature, a crucial aspect for exploiting geothermal resources and designing high-level radioactive waste repositories. Hartmann et al.^14^., Qi et al.^15^., and Pan et al.^16^. emphasize the intimate relationship between mineral composition, density, microstructure, porosity, and heat conductivity in granite. Granite's thermal conductivity is influenced by temperature and water content, with an increase in water content leading to higher thermal conductivity^17,18^. Notably, there is a dearth of studies on the thermal conductivity of high-temperature granite under water cooling^19^, as most studies are conducted on granite in its native state.Despite significant progress in researching the physical characteristics of high-temperature granite, existing independent physical metrics, especially for geothermal extraction, fall short of adequately describing these characteristics. Therefore, there is a critical need to develop a comprehensive index to measure the thermal conductivity of high-temperature granite.

In this paper, The water-cooling test of high-temperature granite was conducted to study the physical properties of granite under different high-temperature treatments. Essential physical parameters at different temperatures were obtained by testing properties such as mass, volume, density, wave velocity, and thermal conductivity. Recognizing the impact of the physical characteristics of granite on geothermal exploitation efficiency, it becomes apparent that a single physical parameter index cannot fully capture the thermal conductivity of reservoirs. Consequently, the Principal Component Analysis (PCA) method was employed to analyze the correlation between physical parameters and derive a comprehensive index reflecting the geothermal exploitation efficiency of granite at different temperatures.

## Basic principle of the PCA method

The complexity of multivariate analysis problems is due to the high dimension, that is, too many related physical parameters are related to each other. Therefore, the main content of principal component analysis is to construct a few comprehensive ones from a large number of indices, which can comprehensively reflect the information of original physical parameters. Besides, the information reflected is not repeated as much as possible.

The petrophysical parameters of the reservoir are defined as *X*.

1 $$X = \left( {x_{1} ,x_{2} ,x_{3} , \cdot \cdot \cdot x_{p} } \right)^{\prime } ,p \ge 2,E\left( X \right) = \mu ,D\left( X \right) = V \ge 0$$

The basic idea of constructing the comprehensive indices of reservoir petrophysical parameters is to find linear combination *y*_1_ of the physical parameters of each component of *X*. It is necessary to make *y*_1_ have the largest variance to make *y*_1_ reflect the changes of *X* as much as possible. Then the second linear combination *y*_2_ of physical parameters of each component of *X* is found.

To make *y*_1_ and *y*_2_ contain no duplicate information as much as possible and reflect the information of *X* as much as possible, it is necessary to make *y*_1_ and *y*_2_ have the maximum variance under irrelevant conditions. According to this method, all the information of *X* is extracted, and *y*_1_ and *y*_2_ as new comprehensive indices are the principal components of *X*.

It is difficult to explain the meaning of its linear combination for the thermal damage problems of granite because of the different units of physical parameters of granite. Physical parameter variables are usually standardized to eliminate the influence of different units among physical parameters (see Eq. (2)).

2 $$X^{*} = \left( {x_{1}^{*} ,x_{2}^{*} , \ldots ,x_{p}^{*} } \right)^{\prime } = \left[ {\frac{{x_{1} - \mu_{1} }}{{\sqrt {\sigma_{11} } }},\frac{{x_{2} - \mu_{2} }}{{\sqrt {\sigma_{22} } }}, \ldots ,\frac{{x_{p} - \mu_{p} }}{{\sqrt {\sigma_{pp} } }}} \right]^{\prime }$$

where $$\mu = E\left( X \right) = \left( {\mu_{1} ,\mu_{2} , \ldots ,\mu_{p} } \right)^{\prime } ,V = D\left( X \right) = \left( {\sigma_{ij} } \right)_{p \times p}$$.

The principle of data standardization is to subtract the mean value from original data and then divide it by the standard deviation. *X* principal component analysis is carried out according to the following steps after data standardization.

1. Find the characteristic root of covariance matrix *V* of *X*, and record it as
  3 $$\lambda_{1} \ge \lambda_{2} \ge \cdot \cdot \cdot \ge \lambda_{k} > 0,\lambda_{k + 1} = \cdot \cdot \cdot = \lambda_{p} = 0$$
2. Find unit eigenvector *μ*_*j*_ corresponding to *λ*_*j*_, and *j* = 1, 2,…, and *k*.
3. *y*_*j*_ = *μ*^'^_*j*_*X,* which is the *j*-th principal component of *X.*

The principal component analysis aims to replace *p*-related variables *x*_1_, *x*_2_,…, and *x*_*p*_ with as few irrelevant principal components *y*_1_, *y*_2_,…, and* y*_*k*_ (*k* ≤ *p*) as possible, and satisfy the statistical characteristics of *X* = (*x*_1_, *x*_2_,…, and *x*_*p*_)'. Then the practical significance of *y*_1_, *y*_2_,…, and *y*_k_ is explained. The number of principal components needs to be determined according to the contribution rate of principal components, which is the ratio of the characteristic roots of the principal components to all the characteristic roots. Principal components with small contribution rates are often omitted in practical applications.

## Evaluation index of the geothermal exploitation efficiency

Geothermal energy mainly occurs in the deep part of the earth. According to the occurrence conditions, it is mainly divided into hydrothermal geothermal energy and dry hot rock geothermal energy. At present, the geothermal energy of dry hot rocks is exploited based on heat exchange technology, which is essentially the rapid cooling of high-temperature reservoir rocks. Geothermal reservoir rocks are highly compact and low permeable, and the rock type is mainly granite.

In order to assess the effectiveness of geothermal exploitation, granite was chosen as the research subject. The change rules of physical properties of granite reservoirs at different temperatures were obtained by cooling granite with water at different temperatures. The correlation between physical parameters was analyzed to propose the evaluation index of comprehensive thermal conductivity of the geothermal reservoir.

### Determination of petrophysical parameters of the geothermal reservoir

**Figure 1:**
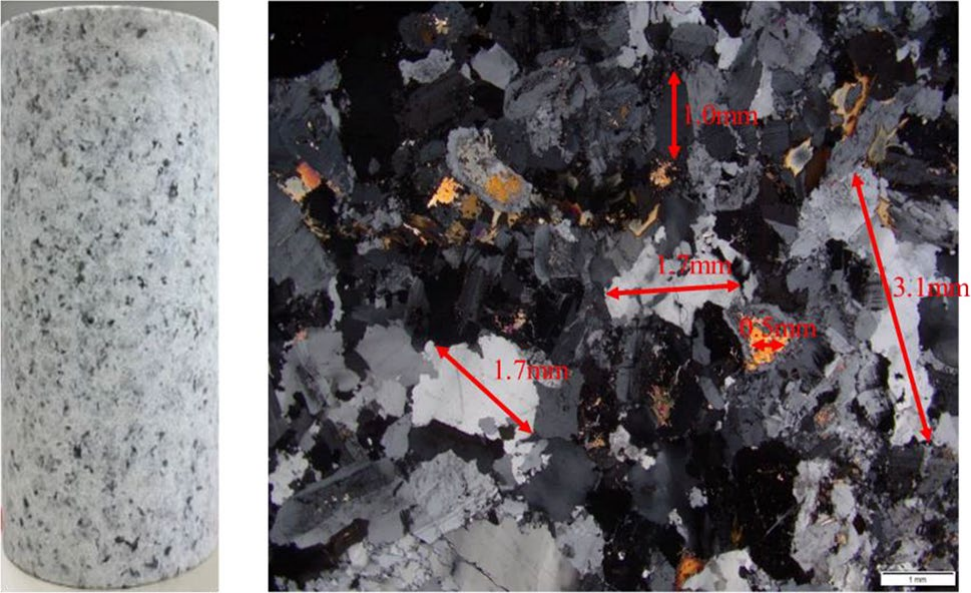
Polarizing microscope results (50 magnification).

**Figure 2:**
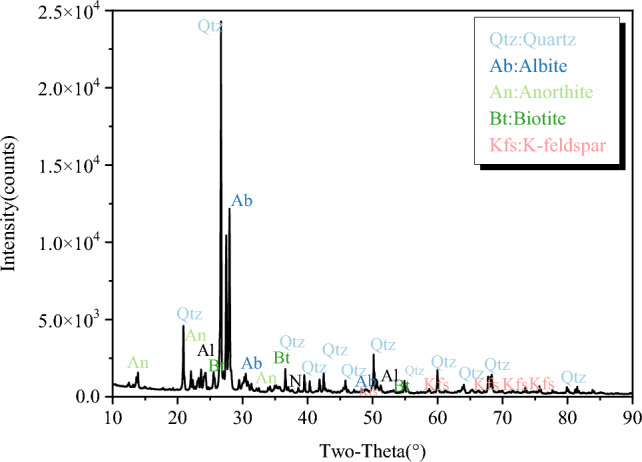
XRD results.

To lessen the spread of test findings caused by the inhomogeneity of the granite specimens, specimens for this test were all collected from the same granite blocks. According to the approach advised by ISRM, the granite specimen had a height-diameter ratio of 2:1, a diameter of NX 54.7 mm, and a length of 100 mm, as shown in Fig. 1. The side surface and two end faces of the granite specimen were polished without obvious defects, and flatness and roughness were controlled within 10 and 3 μm. According to XRD analysis, the main components of granite in this test were quartz, feldspar, biotite, and amphibole, as shown in Fig. 2. It has a uniaxial compressive strength of 216.44 MPa at room temperature, a density of 2,616.48 kg/m^3^, and a porosity of 0.57%.

**Figure 3:**
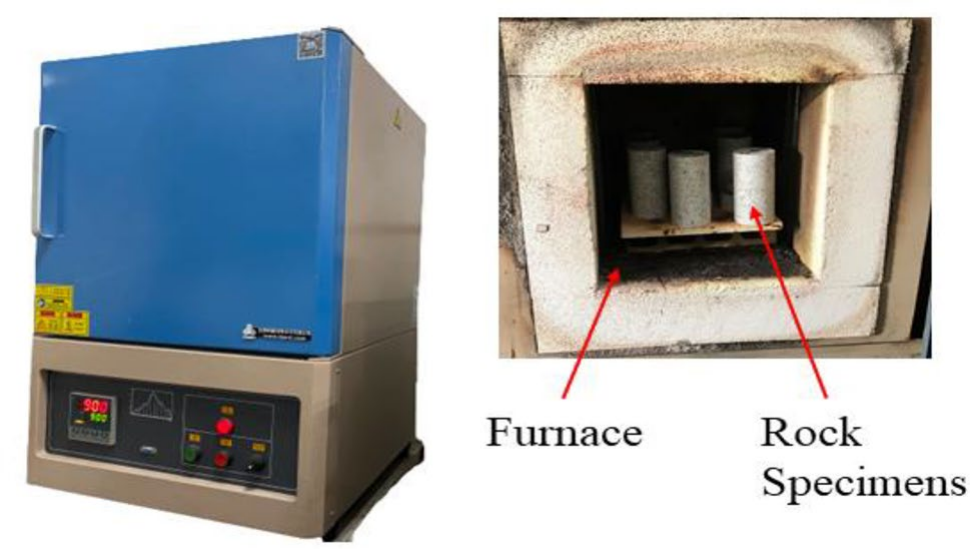
High-temperature furnace.

Granite specimens were meticulously distributed among 8 temperature groups, ranging from room temperatures, to ensure the precision of test data. Within each temperature group, three granite specimens underwent testing, and the final calculations were based on the average values obtained. Firstly, the granite specimen was heated in the high-temperature box muffle furnace. The muffle furnace's temperature control system had an accuracy of 1 °C and a maximum temperature of 1700 °C, which was high enough to pass the test (see Fig. 3). The granite specimens were heated to 150, 300, 450, 600, 750, 900, and 1050 °C at a rate of 2 °C/min, and then maintained at the desired temperature for 2 h.

**Figure 4:**
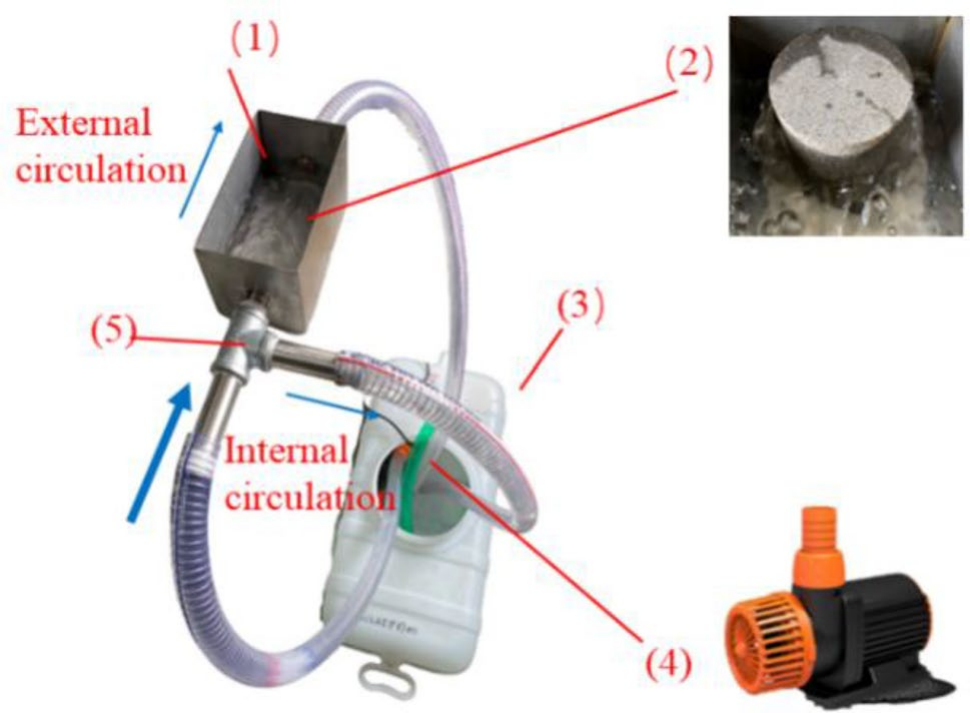
Circulating water system of high-temperature granite: (1) Flume; (2) Granite specimens; (3) Water reservoir; (4) Variable frequency pump; (5) Three-port valve.

Granite specimens were put into the self-made circulating water system (see Fig. 4) for cooling after fully reaching the target temperature. Simulate fluid flow on the high-temperature granite surface for rapid heat exchange and cooling. The self-made circulating water system for high-temperature granite was divided into internal circulation and external circulation. The circulating water exchanged heat with high-temperature granite by external circulation and transferred heat to the water reservoir for the rapid cooling of granite. Water in the water reservoir flowed by internal circulation to consume heat brought by high-temperature granite, without changing the water temperature.

Granite specimens' mass and volume were measured both before and after temperature treatment. A vernier caliper was used to measure the granite specimens' diameter and height, after which the volume was determined. To reduce measurement mistakes, the diameter was measured once every 25 mm from top to bottom. At 120° intervals, the height was measured clockwise, with the final measurement taking the average of the three measurements. Granite specimens were weighed three times with a precision electronic balance before and after thermal treatment, and the average value was finally taken for calculations. Granite specimens cooled by water were dried before the test to ensure that they had not been affected by water attached to the granite specimen surface.

**Figure 5:**
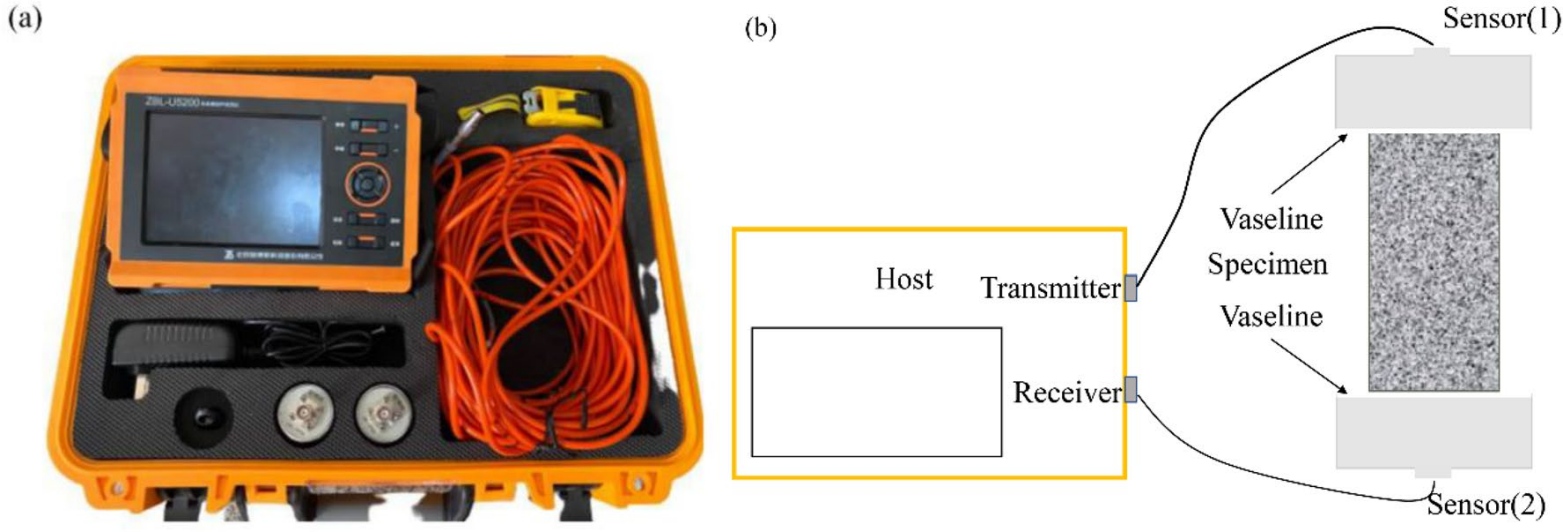
P-wave velocity measurement system: (**a**) ZBL-U5200 acoustic detector; (**b**) Sketch map of P-wave velocity measurement.

To investigate the characteristics of thermal damage in granite specimens, a P-wave test was performed on high-temperature granite specimens with various cooling mechanisms. As the P-wave velocity testing system (see Fig. 5), the ZBL-U5200 non-metallic ultrasonic detector was employed. There was a host, a transmitting end, and a receiving end in the system. Signals of the P-wave were transmitted by the transmitting end and received by the receiving end. To keep the sensor and granite specimen in contact and stop air between them from impacting the test results, a layer of Vaseline was evenly applied to the contact surface.

**Figure 6:**
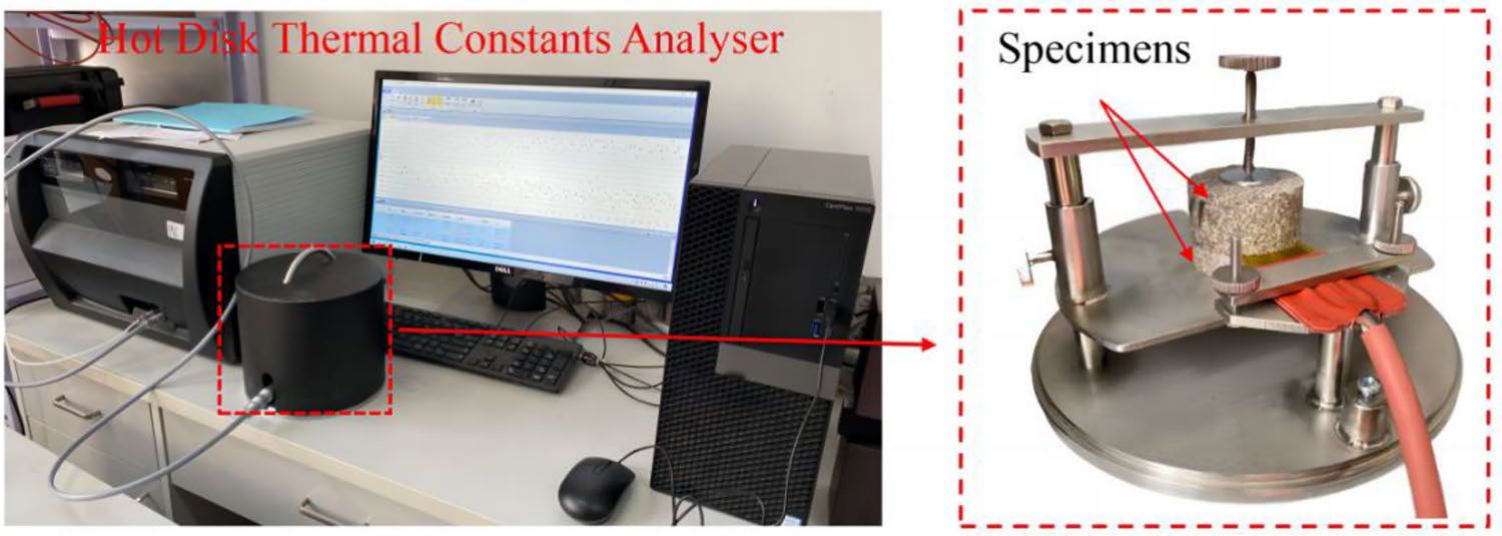
Hot disk TPS 2500 S system.

The thermal constant test equipment (Hot Disk TPS 2500 S) was employed to test the thermal conductivity of granite following thermal treatment using the transient plane source (TPS) method (see Fig. 6). The TPS method can test granite's thermal conductivity, thermal diffusion coefficient, and volume-specific heat capacity following thermal treatment. It is one of the most precise and practical ways to analyze thermal conductivity. The Hot Disk thermal constant analyzer is frequently used to implement the procedure. The photolithographic metal foils (nickel wires) that make up the conductive double helix winding that makes up the Hot Disk sensor.

Thermal conductivity can be measured using the TPS 2500 S system in the range of 0.01–400 W/(m K). Granite specimens can be heated using the hot disk sensor, which can also be used to capture dynamic variations in temperature. According to the relationship between the sensor size and the shape of the granite specimen, the sensor radius selected in this test was 6.40 mm. The granite specimens were installed first. Granite specimens were kept in close contact with the sensor by tightening bolts, and then currents were applied to heat the sensor with constant power. Heat was transferred to the granite specimen after the temperature of the sensor rose. If the granite specimen had strong thermal conductivity, heat would be transferred to the granite specimen rapidly. Heat could not be transferred to the granite specimen for the granite specimen with poor thermal conductivity, and the temperature of the sensor rapidly rose. Therefore, the relationship between temperature rises and time could be recorded by the sensor, and after producing a regression analysis on temperature fluctuations, the thermal conductivity of rock specimens was discovered.

### Evolution rule of physical parameter indices with temperatures

The mass loss of the granite specimens was caused by changes in interior moisture after thermal treatment. In addition, the volume change was accompanied by an increase in the mass loss due to the growth of microcracks and the loss of mineral particles brought on by the expansion and contraction of mineral particles.

The values for granite specimens' mass, volume, and density before and after treatment at various temperatures are displayed in Table 1. *K*_*m*_ (mass-loss rate), *K*_*v*_ (volume-increase rate), and *K*_*ρ*_ (density-change rate) are three variables that can be used to indicate changes in mass, volume, and density. *K*_*m*_ stands for the mass loss/initial mass ratio; *K*_*v*_ for the volume increase/initial volume ratio; *K*_*ρ*_ for the density change/initial density ratio; and *K*_*N*_ for the rate of change of physical parameters.

4 $$K_{N} = \frac{\Delta N}{{N_{0} }} \times 100\%$$

where *△N* is the variation of physical parameters; *N*_0_ the initial value of physical parameters.

**Table 1:** Relevant physical parameters of specimens before and after thermal treatment.

Group	Before thermal treatment (25 °C)				After thermal treatment				
	Mass (g)	Volume (cm^3^)	Density (kg/m^3^)	P-wave velocity (km/s)	Temperature ( °C)	Mass (g)	Volume (cm^3^)	Density (kg/m^3^)	P-wave velocity (km/s)
1	513.48	196.25	2616.48	4.36	25	513.48	196.25	2616.48	4.36
2	512.73	195.66	2620.50	4.25	150	512.51	195.60	2620.24	3.39
3	512.94	195.21	2627.70	4.28	300	512.55	196.45	2608.99	2.88
4	512.83	195.53	2622.76	4.30	450	512.13	197.62	2591.53	2.19
5	512.98	195.73	2620.90	4.20	600	511.55	202.71	2523.60	1.34
6	514.20	195.53	2629.76	4.60	750	512.45	203.40	2519.38	1.00
7	513.25	195.86	2620.53	4.36	900	510.81	206.74	2470.79	0.76
8	513.61	195.79	2623.22	4.20	1050	510.49	213.67	2389.11	0.34

**Figure 7:**
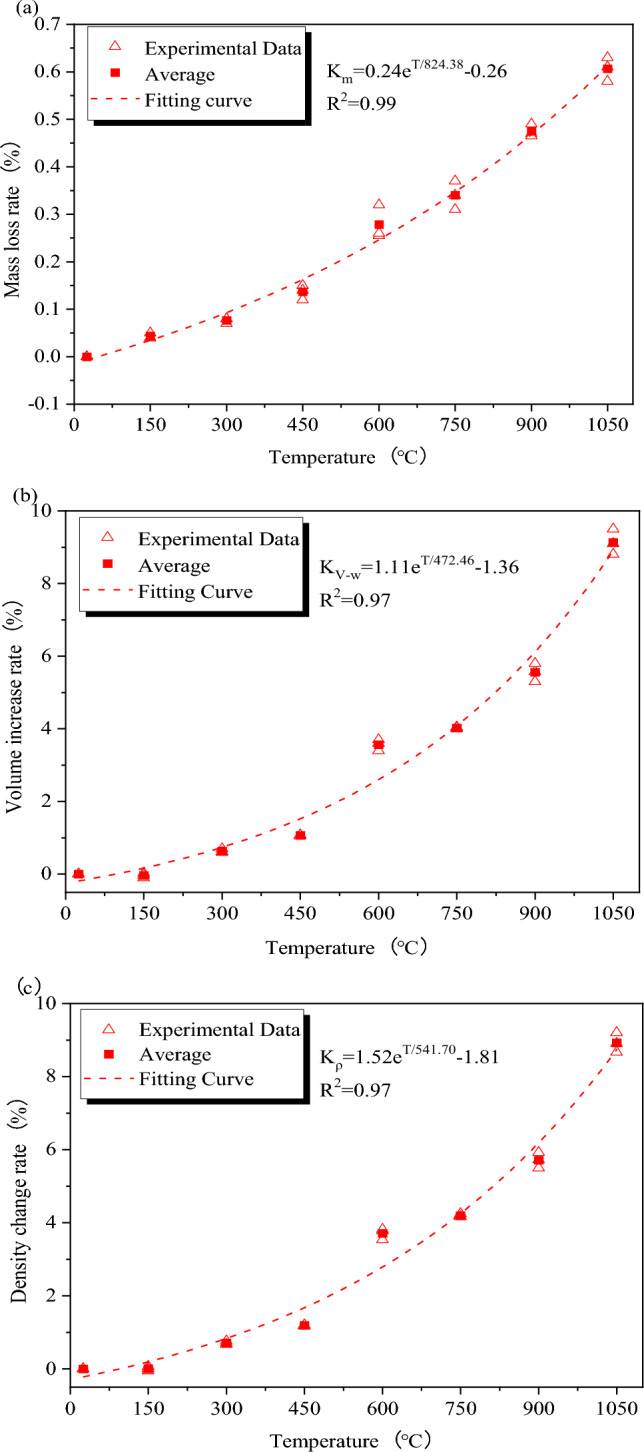
Change rates of relevant physical parameters of granite specimens after different thermal treatments: (**a**) Mass loss rate; (**b**) Volume increase rate; (**c**) Density change rate.

Figure 7 depicts the modifications to granite specimens' mass, volume, and density following various thermal treatments. For discussion, the temperature change curve from 25 to 1050 °C is broken down into four sections. The density change rate was unchanged at the first stage (25–150 °C), and the overall structure of the granite specimen remains. The density change rate increases rapidly at the second stage (150–600 °C), and the overall structure of the granite specimen becomes loose rapidly. The density change rate increases slowly at the third stage (600–750 °C), and the structural looseness of granite specimens increases slowly. The density changes most obviously with the temperature at the fourth stage (750–1050 °C). The growth rate of changes is the largest, and the looseness of granite specimens reaches a peak.

Granite has the ability to conduct acoustic waves, and the porosity, particle cementation level, and composition of the granite all have an impact on the P-wave's velocity. Therefore, determining the P-wave velocity is crucial for identifying and assessing granite damage. Wave velocity attenuation rate *K*_*P*_, which is the ratio of wave velocity attenuation to the starting wave velocity, can be used to express the fluctuation of P-wave velocity of thermally damaged granite. In this test, the granite specimen's average wave velocity is 4,360 m/s before thermal treatment. According to Table 1, the P-wave velocity attenuation rate represents the degree of damage to the granite after thermal treatment. Wave velocity change data of granite specimens before and after various thermal treatments are determined.

**Figure 8:**
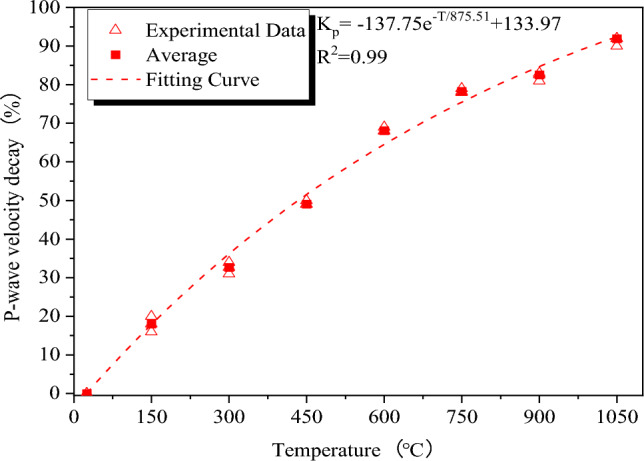
P-wave velocity decay rates of granite specimens after different thermal treatments.

The fluctuation of P-wave velocity at various temperatures is depicted in Fig. 8. There are four stages to the evolution of wave velocity attenuation rate. Because more bound and free water vaporizes at higher temperatures to create more voids, the attenuation rate of the wave velocity increases linearly with temperature at the first stage of 25–300 °C.

The wave velocity attenuation rate continues to increase and reaches 68.09% at the second stage of 300–600 °C, indicating that granite specimens are seriously damaged. Firstly, quartz in granite undergoes an Alpha Beta Quartz transition at 573 °C, followed by crystal expansion and movement, leading to the development of internal fractures in the rock. Brittle rocks undergo a process of crack development and transform into plastic rocks, which affects the propagation of P-waves. When the space is not enough for particles to expand, thermal stress damages the connection between the particles and weakens the cementation degree. New cracks are generated around the particles^20^, and the damage caused by thermal stress is irreversible. Particles cooled by water shrink rapidly and produce microcracks again, which causes the looser arrangement of particles and increased voids and hinders the propagation of P-waves in granite.

The attenuation rate of the wave velocity slows down at the third stage of 600–750 °C mainly because the temperature causes less damage to granite. The wave velocity attenuation rate increases at the fourth stage of 750–1050 °C. Some new microcracks form in granite specimens due to decreased water cooling, and under both cooling techniques, granite tends to attenuate waves at rates that are similar.

The effectiveness of geothermal resource extraction greatly depends on the granite found in geothermal reservoirs' thermal conductivity. The TPS method was used to assess the thermal conductivity and diffusivity of granite under various thermal treatment settings, and it was also used to look at how temperature and cooling modes affected granite specimens' thermal conductivity.

**Figure 9:**
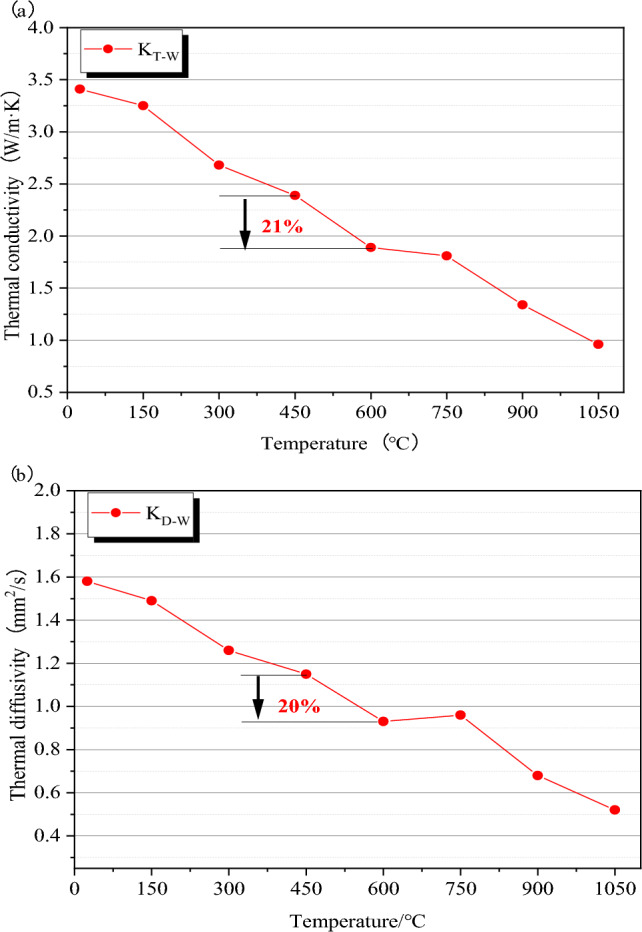
Thermal conductivity characteristics of granite specimens after different thermal treatments: (**a**) Thermal conductivity; (**b**) Thermal diffusivity.

Based on the test results of the mass, volume, and wave velocity of granite specimens, statistical analysis showed a small standard deviation of results. The granite specimens were good homogeneous before thermal treatment and showed certain rules after thermal treatment. The mass, volume, and wave velocity tests were non-destructive. The average thermal conductivity of the granite specimen was taken in the following tests (see Table 2 for the test results of thermal conductivity). Prior to thermal treatment, granite test specimens had an average thermal conductivity of 3.41 W/(mK). Granite specimens' *K*_*T*_ and *K*_*D*_ values were used to express, respectively, their thermal conductivity and thermal diffusivity. The variations of *K*_*T*_ and *K*_*D*_ with thermal treatment temperature are shown in Fig. 9.

**Table 2:** Thermal conductivity of granite specimens after thermal treatments.

Temperature ( °C)	Thermal conductivity (W/(m K))			Thermal diffusivity (mm^2^/s)		
25	3.35	3.43	3.44	1.47	1.62	1.65
150	3.29	3.21	3.25	1.55	1.49	1.59
300	2.70	2.68	2.65	1.27	1.25	1.24
450	2.41	2.38	2.39	1.17	1.14	1.12
600	1.90	1.89	1.88	0.94	0.93	0.93
750	1.81	1.81	1.80	0.99	0.95	0.94
900	1.36	1.33	1.33	0.71	0.66	0.66
1050	0.97	0.96	0.96	0.53	0.52	0.52

Granite specimens' thermal conductivity and diffusivity both exhibited non-linear declining trends as the thermal treatment temperature was raised. The change rules tended to be similar, and factors affecting their changes were roughly the same. *K*_*T*_ changed a little from 25 to 150 °C by taking the variation law of thermal conductivity as an example, indicating that the escape of a small amount of free water had little effect on the thermal conductivity. *K*_*m*_ and *K*_*V*_ rarely changed, and *K*_*p*_ rose slightly due to the influence of the void ratio. *K*_*T*_ decreased gradually from 150 to 450 °C. Combined water escaped through gasification, which formed many gaps; then the microcracks caused by thermal stress developed. Granite specimens developed a variety of microcracks, which grew wider and more numerous as the temperature rose.

The quick drop in *K*_*T*_ and the maximal deceleration rate at 450–600 °C indicate that the impact of microcracks on thermal conductivity increased. Thermal stress caused microcracks that acted as a barrier to heat transport, drastically lowering thermal conductivity. The temperature threshold might be thought of as 450 °C. *K*_*m*_, *K*_*V*_, and *K*_*p*_ all increased dramatically, while *K*_*T*_ changed quickly. Combined water evaporated rapidly, and the density and diameter of the microcracks both grew with time. Granite's thermal conductivity was altered by the phase transformation of quartz^21–23^.

*K*_*T*_ continued to decrease at 600–750 °C, but the decay rate decreased, indicating that thermal treatment further led to the microcrack development. However, the number of new microcracks turned out to be small. The increase rates of *K*_*m*_, *K*_*V*_, and *K*_*p*_ slowed down, and the changing trend was similar to that of *K*_*T*_. *K*_*T*_ decayed rapidly at 750–1050 °C because quartz particles changed from β—state quartz to β—state tridymite at 870 °C. The volume of quartz particles increased by 16%.

## Comprehensive index analysis of geothermal exploitation

Research on comprehensive indices of geothermal exploitation belongs to multivariate analysis. The main content of multivariate analysis is to discuss the correlation between vector components. In other words, it is a study of the correlations between physical parameter values under different/same temperatures. Many physical parameters of thermally damaged granite are related. Multivariate correlation analysis can find a comprehensive index to describe whole granite. Principal component analysis (PCA) and canonical correlation analysis (CCA) are two often employed statistical techniques for multivariate correlation analysis. The PCA approach is employed in this part because canonical correlation analysis is used to examine the interdependence between two vectors.

The original data of physical parameters of granite specimens cooled by water under different temperatures could be obtained from Tables 1 and 2. Data analysis was performed using a computational tool, and the PCA method was applied for calculations. The original data were standardized to eliminate the influence of different physical parameter units on the principal components, resulting in dimensionless data (see Table 3).

**Table 3:** Standardized treatment of physical parameters of granite specimens.

Temperature ( °C)	Mass (*m*)	Volume (*V*)	Density (*ρ*)	P-wave velocity (*P*)	Thermal conductivity (*K*_*T*_)	Thermal diffusivity (*K*_*D*_)
25	1.4976	− 0.8335	0.8941	1.6439	1.3931	1.3270
150	0.5186	− 0.9357	0.9396	0.9588	1.1775	1.2303
300	0.5589	− 0.8021	0.8036	0.5986	0.4965	0.4658
450	0.1350	− 0.6183	0.5925	0.1112	0.2015	0.1758
600	− 0.4504	0.1815	− 0.2286	− 0.4891	− 0.3774	− 0.3779
750	0.4580	0.2890	− 0.2797	− 0.7292	− 0.4682	− 0.3076
900	− 1.1973	0.8147	− 0.8670	− 0.8987	− 1.0016	− 1.0546
1050	− 1.5203	1.9035	− 1.8544	− 1.1954	− 1.4215	− 1.4588

Subsequently, a computational tool was employed to calculate standardized data following steps (5)–(7) of the PCA method, and the six characteristic roots of the characteristic equation were determined. The transformation matrix (eigenvector) of the principal components is obtained as.

5 $$\begin{gathered} \lambda_{{1}} = {5}.{6354},\lambda_{{2}} = 0.{1924},\lambda_{{3}} = 0.{1493},\lambda_{{4}} = 0.0{227},\lambda_{{5}} = 0.000{2},\;{\text{and}}\;\lambda_{{6}} = 0.000{1} \hfill \\ \left| {\begin{array}{*{20}c} {0.3940} & {0.1721} & {0.8936} & { - 0.1182} & { - 0.0492} & {0.0169} \\ { - 0.4063} & {0.5934} & {0.1027} & {0.1282} & {0.5245} & {0.4250} \\ {0.4101} & { - 0.5146} & { - 0.0743} & { - 0.1334} & {0.6021} & {0.4257} \\ {0.4045} & {0.5119} & { - 0.3518} & { - 0.6368} & {0.1072} & { - 0.1834} \\ {0.4169} & {0.2349} & { - 0.2253} & {0.3176} & { - 0.4810} & {0.6229} \\ {0.4173} & {0.1903} & { - 0.1048} & {0.6674} & {0.3421} & { - 0.4650} \\ \end{array} } \right| \hfill \\ \end{gathered}$$

The contribution rate of the characteristic roots is calculated to obtain as few uncorrelated principal components as possible.

6 $$\frac{5.6354}{{5.6354 + 0.1924 + 0.1493 + 0.0227 + 0.0002 + 0.001}} = 93.9233\%$$

When the first principal component is retained, the cumulative contribution rate has reached 93.9233%. It is sufficient to meet the statistical requirements if the total contribution rate of the first *n* main components reaches 85%. Therefore, the expression of the principal components of the physical parameters of thermally damaged granite is obtained as Eq. (7) that the signs of coefficients have five positive (*m*, *ρ*, *P*, *K*_*T*_, and *K*_*D*_) and a negative (*V*).

7 $$y = 0.3940m - 0.4063V + 0.4101\rho + 0.4045P + 0.4169K_{T} + 0.4173K_{D}$$

When the *y* value of thermally damaged granite is large, *m*, *ρ*, *P*, *K*_*T*_, and K_*D*_ are large, but *y* is small. That is, the granite specimen has high density, small volume, complete granite structure, and good thermal conductivity. For the exploitation of geothermal resources, the *y* value can be used as a symbol of the comprehensive thermal conductivity of granite reservoirs as well as one of the comprehensive indices to evaluate the exploitation efficiency of geothermal reservoirs.

**Figure 10:**
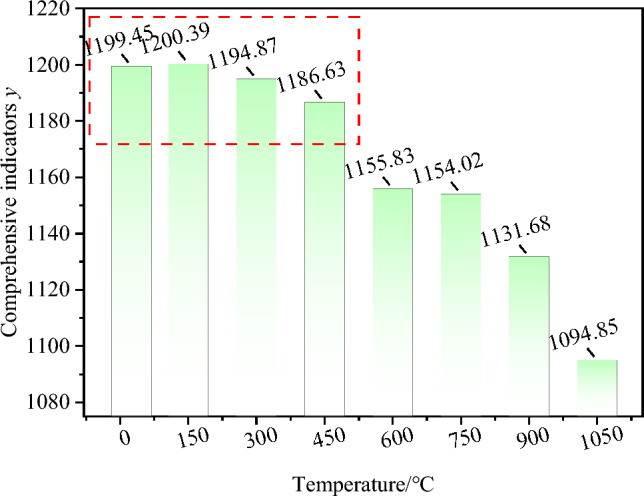
Comprehensive indices of geothermal resource exploitation under different temperatures.

Granite specimens with 8 temperature gradients are sorted according to the y expression from large to small, 150 °C > 300 °C > 450 °C > 600 °C > 750 °C > 900 °C > 1050 °C. Figure 10 shows the changing relationship between *y* and the increased temperature. y decreases with the increased temperature at 150–450 °C, but the decline rate is quite slow and even can be neglected in the whole change process at 150–1050 °C. When the temperature exceeds 450 °C, the y value decreases rapidly. The changed *y* value affected the thermal conductivity of the geothermal reservoir seriously. Moreover, the increased temperature reflects the increased granite occurrence depth, which hinders geothermal good construction. According to the comprehensive analysis, 150–450 °C is more suitable for geothermal resource exploitation.

## Discussions

Thermal treatment can modify the physical properties of granite by analyzing the rule that the physical properties of thermally damaged granite change with the increased thermal treatment temperature. The density, P-wave velocity, and thermal conductivity corresponding to each group of temperatures in the test data are normalized to calculate the modification coefficient under each temperature state and analyze the modification effect of different physical parameters under thermal treatment and the sensitivity of different physical parameters under the same cooling mode.

8 $$m = \frac{{N_{T} }}{{N_{0} }}$$

where *m* is the modification coefficient of granite under thermal treatment; *N*_*T*_ the physical parameter under different temperatures; *T* the temperature of thermal treatment; *N*_*0*_ the physical parameter in a natural state.

**Figure 11:**
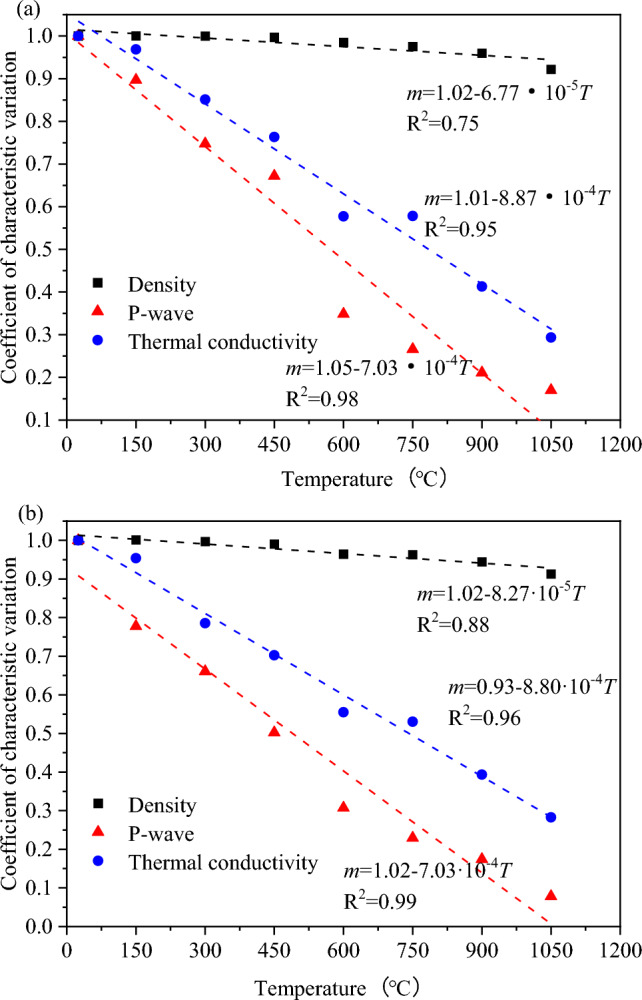
Fitting of the modification effect of granite’s physical parameters under different thermal treatments: (**a**) air-cooling; (**b**) water-cooling.

Without any thermal treatment, granite is used as a specimen in its original state. The modification coefficients of density, P-wave velocity, and thermal conductivity of the granite specimen in its natural state are 1. The change in density, P-wave velocity, and thermal conductivity of the granite specimen with the rising temperature is an attenuation process, according to the evolution of physical attributes of thermally-damaged granite. As a result, at different temperatures, the modification coefficients for density, P-wave velocity, and thermal conductivity are all less than 1. The modification effect of physical characteristics is less pronounced the closer the modification coefficient is near 1. Tables 1 and 2 provide information on granite's density, P-wave velocity, and thermal conductivity at various temperatures. The modification coefficients of the physical characteristics of granite at various temperatures are calculated by Eq. (8) (see Fig. 11).

The change curve of physical parameters with temperatures is linearly fitted to establish the functional expression of modification coefficients between the thermal treatment temperature and physical parameters of granite. Therefore, modification coefficients between the temperature and physical parameters meet the linear-function relationship.

9 $$m = a - bT$$

where *a* and *b* are fitting constants.

The modification coefficients of the physical parameters of granite after the thermal treatment decreases linearly with the increased temperature (see Fig. 11). The modification coefficients of the granite specimen after water cooling are smaller than those after air cooling. The P-wave velocity > thermal conductivity > density by comparing the sensitivity of physical parameters with the increased temperature. The P-wave velocity of thermally damaged granite responds most rapidly to the temperature, and the wave velocity test is simple to operate and wide in scope. Besides, it does not damage granite. Therefore, longitudinal-wave-velocity detection can be used for thermally damaged granite. The geothermal-well temperature can be retrieved in geothermal resource exploration by detecting the wave velocity of the core drilled from the geothermal well.

## Conclusions

Investigating the temperature-induced changes in properties such as mass loss rate, volume increase rate, wave velocity decay rate, and thermal conductivity in thermally damaged granite specimens revealed essential correlations in their physical characteristics. The study's key findings are summarized as follows:

1. Exponential changes in mass loss rate, volume expansion rate, and density were observed with increasing temperature. The dominant factor at 25–150 °C was the escape of free water, shifting to thermal stress at 150–600 °C. Beyond 450 °C, a significant increase in the change rate occurred due to combined water and thermal stress. At 600–750 °C, thermal stress remained dominant, with a subsequent phase transformation at 750–1050 °C.
2. The attenuation rate of the wave velocity increased progressively with rising temperature. Gradual acceleration occurred at 25–300 °C, continuous increase at 300–600 °C, and a slowdown at 600–750 °C. A significant rise at 750–1050 °C indicated severe damage to the granite.
3. The thermal conductivity of thermally damaged granite exhibited temperature dependence. A non-linear decrease was noted from 25 to 1050 °C after thermal treatment, peaking at 450–600 °C. At 1050 °C, the thermal conductivity was only one-third of the initial value.
4. A PCA-based correlation model between granite reservoir physical parameters was established. '*y*' was proposed as the comprehensive index for the thermal conductivity of granite reservoirs. The temperature range of 150–450 °C was identified as suitable for geothermal exploitation. An evaluation method for the modification effect of physical parameters in thermally damaged granite was proposed, with priority given to using P-wave velocity for detecting thermal damage.

## Data Availability

Data will be made available on request. The first author should be contacted if someone wants to request the data from this study.
